# Alleviation of Plaque and Gingivitis with Dental Water Jet in Regular and Orthodontic Patients: A Systematic Review and Meta-Analysis

**DOI:** 10.3390/healthcare13040396

**Published:** 2025-02-12

**Authors:** Arwa Badahdah, Murooj Abdulrahim Hariri, Modi Salman Aljohani, Layan Saad Alshehri, Zuhair S. Natto

**Affiliations:** 1 Department of Periodontics, Faculty of Dentistry, King Abdulaziz University, Jeddah 21589, Saudi Arabia; 2 Restorative Dentistry Department, King Fahad General Hospital, Jeddah 23325, Saudi Arabia; muroojhariri@gmail.com; 3 King Abdulaziz Medical City, Jeddah 21423, Saudi Arabia; modisalmanjohani@gmail.com; 4 Endodontic Resident, Faculty of Dentistry, King Abdulaziz University, Jeddah 21589, Saudi Arabia; layan.alshehri2@gmail.com; 5 Department of Dental Public Health, Faculty of Dentistry, King Abdulaziz University, Jeddah 21589, Saudi Arabia; znatto@kau.edu.sa

**Keywords:** bleeding index, flossing, gingival index, interproximal cleaning, irrigation, periodontal diseases, plaque, waterjet

## Abstract

**Objectives:** This review aimed to compare the effectiveness of using a dental water jet (WJ) to manual tooth brushing (MTB) alone or when combined with flossing (MTB + F) in improving plaque index (PI), bleeding index (BI), and gingival index (GI) in regular and orthodontic patients. **Materials and Method:** This review was registered with the PROSPERO registry (CRD42022296752). Three reviewers conducted a comprehensive search of MEDLINE, the Cochrane Library, and Google Scholar for studies published between 1990 and July 2022. Eligible studies were randomized clinical trials, excluding those involving peri-implantitis, patients with impaired manual dexterity, or powered brushes. The outcomes of interest (PI, BI, and GI) were measured across short-, intermediate-, and long-term periods. **Results:** Eighteen RCTs were included in this review. PI: in regular patients, use of a WJ showed no additional improvement over flossing, and the results were inconsistent when compared to MTB alone. For orthodontic patients, use of a WJ provided a slight improvement over flossing in the short term and MTB alone in the intermediate term. BI: use of a WJ demonstrated a slight improvement over flossing and MTB alone in both patient groups at different time points. GI: use of a WJ was comparable to flossing but showed slight benefits over MTB alone in the short term for regular patients and in the intermediate term for orthodontic patients. **Conclusions:** use of a WJ may provide slight benefits in BI and GI compared to flossing or MTB alone, especially for orthodontic patients. **Clinical relevance:** incorporating a WJ into the daily oral hygiene routine is recommended due to its potential benefits over brushing alone and its user-friendly alternative to flossing.

## 1. Introduction

Evidence showed that the accumulation of dental plaque results in gingivitis. Loe demonstrated a cause–effect relationship between gingivitis and undisturbed dental plaque [1]. Plaque removal using proper oral hygiene measures restores the healthy gingiva and re-establishes normal microflora [2]. Although the multifactorial etiopathogenesis of periodontitis includes periodontal pathogens and host factors, dental plaque is the primary etiological factor [3,4]. A direct relationship between plaque accumulation and periodontal loss has not been established in clinical trials; however, in epidemiological studies, periodontitis is more prevalent in populations with poor oral hygiene. The first National Health and Nutrition Examination Survey (NHANES I) showed a higher prevalence of periodontitis and poor oral hygiene in blacks and males [5]. Plaque control is crucial for the treatment of periodontitis and the maintenance of periodontal health or a stable condition, which is possible for patients who are willing to maintain proper plaque control [6]. In several studies, the absence of plaque was a relatively highly specific predictor of periodontal stability [7,8]. Therefore, oral hygiene education and training are integral for treating periodontitis and periodontal maintenance protocol [9].

Research suggests that eliminating dental plague every two days is usually enough to avoid gingivitis; nevertheless, regular cleaning is necessary for those who have developed gingivitis to control inflammation and preserve oral health [10,11]. Brushing not only removes plaque but also facilitates fluoride application and gets rid of food particles, which is why brushing twice a day is advised [12]. Although no brushing technique has been proven to be better, the modified Bass method is the most commonly recommended since it is the most successful at reaching the gingival sulcus [13,14]. Any brushing technique’s success ultimately depends on the user’s ability to employ it consistently and correctly. When it comes to powered toothbrushes, there is a significant discrepancy among studies regarding their efficacy in plaque removal compared to manual brushing. Two main powered toothbrushes are available: oscillating-rotating brushes with round brush heads and sonic-powered brushes. Oscillating-rotating toothbrushes provide the strongest evidence for reducing plaque and gingivitis, according to a Cochrane systematic review and another network meta-analysis [15,16]. Although powered toothbrushes have long been advised for people with impairments and their caregivers, they may also benefit the general population, particularly those who struggle with manual dexterity or brushing procedures [12].

While toothbrushing is essential for maintaining oral health, it primarily cleans accessible tooth surfaces and does not guarantee sufficient cleaning between the teeth. The effects of interproximal cleaning on gingival health have been extensively studied and, to various extents, support the positive impact of different interproximal devices on gingival health [17,18,19]. A nationwide study in Australia found an association between regular interproximal cleaning and reduced plaque, calculus, and moderate-to-severe gingivitis, but not attachment loss [20]. The European Federation of Periodontology workshop on “Primary prevention of periodontitis: managing gingivitis” recommended once-daily interproximal cleaning using an interdental brush (IDB), which has the highest effectiveness for plaque removal, as the first choice. Given the inconsistent evidence of plaque removal and gingival health, other dental aids are advisable only when atraumatic IDB use is unachievable [21].

The oral irrigator, introduced in the 1960s, is a commercially popular interproximal device [22], with proven safety [23,24], that has been evaluated in numerous clinical trials for plaque removal and improved gingival health. In most studies, irrigators showed no additional benefit in plaque removal compared to flossing [25,26,27] or brushing alone [28,29,30,31], although a few studies demonstrated a superior effect of irrigators [32,33,34,35]. In contrast, studies have increasingly shown the significant benefits of waterjets on gingival health [25,26,32,34,36].

Although several systematic reviews have evaluated oral irrigators or waterjets (WJs), previous reviews have been limited in scope, either by the number of trials included or by a lack of subgroup analyses based on patient type and timeframes. In this systematic review and meta-analysis, we aimed to evaluate the efficacy of dental WJs as an adjunct to manual tooth brushing (MTB) in reducing plaque and improving gingival health by assessing plaque index, gingival index, and bleeding index in both regular and orthodontic patients. Our analysis included a broad range of comparisons, evaluating WJs against both MTB alone and MTB with flossing, while also stratifying outcomes across three distinct follow-up periods: the short, intermediate, and long term.

## 2. Materials and Methods

The PICO elements were meticulously constructed to answer our research question: Is the use of WJ superior to flossing or MTB alone in reducing PI, BI, and GI? The population (P) under study included regular patients and patients with fixed orthodontic appliances. Intervention (I) involved the use of WJ with MTB, whereas the control (C) group used floss with MTB or MTB alone. The outcomes (O) of interest were PI, GI, and BI in the short, medium, and long term, respectively. This review was registered with the PROSPERO registry (CRD42022296752) and strictly adhered to the PRISMA guidelines [37].

### 2.1. Search Strategy

Three reviewers (L.S., M.J., and M.H.) conducted a comprehensive search of three databases independently: MEDLINE (through PubMed), the Cochrane Library, and Google Scholar. The search covered studies published from 1990 to July 2022, with the final search being conducted at the end of July 2022. By combining keywords and Boolean operators “OR” and “AND”, we ensured a comprehensive search across the different databases. The list of keywords used in the search is presented in Supplementary Box S1, and the complete search strategy for MEDLINE (PubMed) is provided in Supplementary Box S2. After the initial search, the same reviewers screened the titles of the initial search results to select eligible studies for an abstract-level review, followed by a full-text review, which was then revised by A.B. and Z.N. In addition to the electronic search, references from all reviews, systematic reviews, and meta-analyses were manually screened for eligible studies to further enhance the comprehensiveness of the search process.

### 2.2. Eligibility Criteria

The eligibility of the studies for inclusion in this analysis was based on the following criteria: (1) randomized clinical trial (RCT); (2) conducted on regular patients or orthodontic patients; (3) reported outcomes including G.I., P.I., or B.I.; (4) use of a home irrigation device in the intervention group; (5) use of floss or only MTB in the control group; and (6) the timing for outcome measurement was two weeks or longer. The exclusion criteria were as follows: (1) trials on patients with peri-implantitis; (2) trials on patients with any form of impaired manual dexterity or uncontrolled systemic conditions that affect periodontal health; (3) use of in-office irrigation only or irrigation with medications or mouthwash; (4) use of powered interproximal devices other than water irrigation; (5) use of a powered brush rather than manual; and (6) use of interproximal aids other than flossing in the control group.

### 2.3. Data Extraction

After selecting the final papers for this study, three reviewers (L.S., M.J., and M.H.) extracted the data for analysis using an Excel spreadsheet, which was then revised by (A.B.). The following data were collected.

- Author, date, study design, and setting.
- Characteristics of the participants: their number, age, and sex.
- Management of the intervention group and the type of water irrigator used.
- Management of the control group.
- Outcomes measured: PI, BI, and GI.

### 2.4. Data Synthesis and Statistical Analysis

To synthesize the findings of the included studies, RevMan software was used (Version 5.4. Copenhagen, Denmark: Nordic Cochrane Centre, The Cochrane Collaboration, 2020). As our data were continuous, we expressed the results as the mean difference (MD) with a 95% confidence interval (CI). Depending on the heterogeneity of the included studies, fixed- and random-effects models were considered. The fixed-effects model was used if all included studies shared a common effect size with any observed differences being due to sampling errors alone. The random-effects model considers both intra- and inter-study variability, allowing for a more conservative estimate of the overall effect size. All analyses and publication biases were assessed at short-, intermediate-, and long-term follow-up intervals by using funnel plots and statistical tests, such as Egger’s regression test and Begg’s rank correlation test. Heterogeneity among studies was assessed using the I^2^ statistic. The thresholds for interpreting the I^2^ values for heterogeneity were as follows: low (25%), moderate (50%), and high (75%). Sensitivity analysis was used to assess the results’ robustness by excluding studies with extreme effect sizes or high risk of bias. This analysis aimed to assess the impact of individual studies on the overall findings and ensure the reliability and validity of the systematic review results.

### 2.5. Quality Assessment

The risk of bias in the included studies was assessed by A.B. and Z.N. using the Cochrane Risk of Bias 2 (ROB 2) tool, which includes five main domains. These domains are “bias in the process of randomization, deviation from intended intervention, missing outcome date, measurement of the outcome, and selection of the reported results” [38]. The judgment options of high risk of bias, low risk, or some concern in each domain were reached using signaling questions provided with the ROB 2 tools.

### 2.6. Assessment of Certainty of Evidence

We applied the GRADE method to evaluate the degree of confidence in the evidence that was provided. We produced “summary of findings” tables based on the GRADE system for the following results: PI, BI, and GI. When assessing the certainty of the evidence, the directness of the evidence, the consistency of the results, the precision of the estimations, and the risk of publication bias were considered. The certainty of the body of evidence was categorized into 4 levels: high, moderate, low, and very low [39,40].

## 3. Results

### 3.1. Search Results

**Figure 1:**
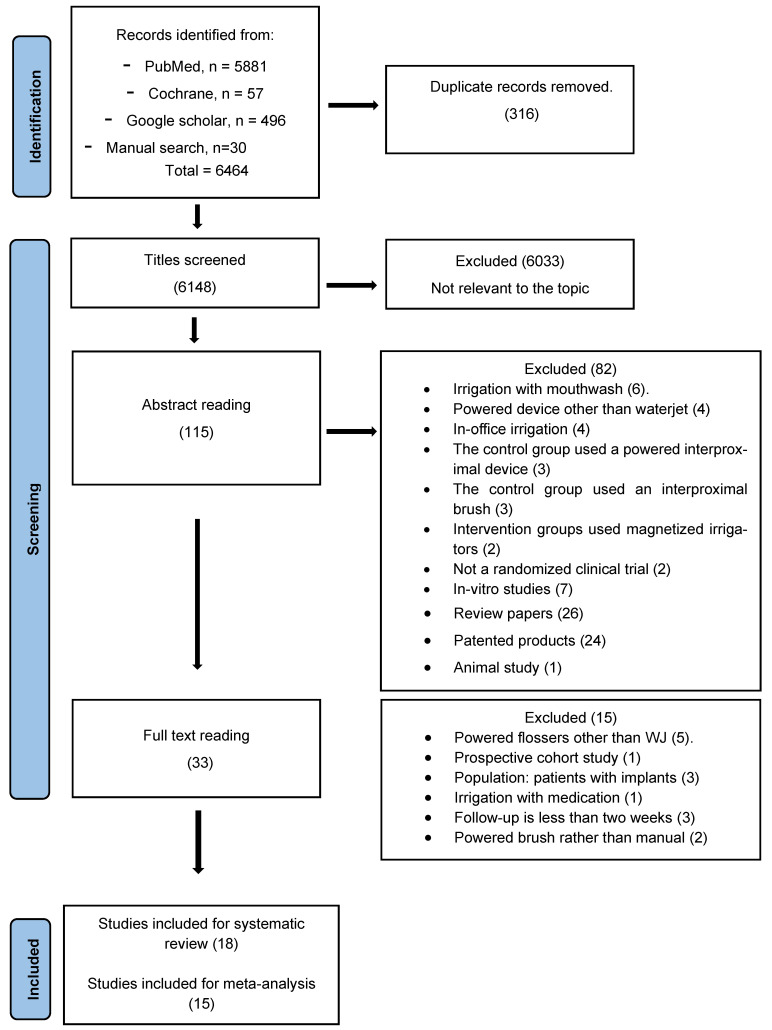
Flow diagram of the search results and selection of the studies.

The initial search of all combined databases revealed 6464 papers. The manual search revealed an additional 30 papers. After removing duplicate papers, 6148 titles were screened for relevance. After title-level exclusion, the abstracts of 115 articles were reviewed, and an additional 82 papers were excluded. Finally, the full texts of 33 studies were reviewed for eligibility, and 18 were selected for inclusion in this systematic review. For the meta-analysis, a quantitative analysis was conducted on 15 studies (Figure 1).

### 3.2. Characteristics of the Included Studies

Table 1 summarizes the study design, sample size, age, number of participants, type of intervention, and measured outcomes. All the studies were RCTs of parallel design except three: one had a crossover design [31], and two had split-mouth designs [27,41]. The samples ranged from 20 to 117 patients, with 1005 participants across all the included groups. Some groups in the included studies were excluded for the following reasons: use of mouthwash or medication for irrigation, professional in-office irrigation, use of powered brushes, or no oral hygiene control. Participants’ ages ranged from 18 to 75 years in studies conducted on non-orthodontic patients. Children were involved in orthodontic studies, and the age range of the participants was 11 to 48 years (Table 1).

### 3.3. Intergroup Comparison

Studies were categorized according to the population involved and the interproximal protocol applied to the intervention and control groups (Table 1).

- Group A comparison: four studies compared MTB + WJ to MTB + floss on regular patients [25,26,42,43].
- Group B comparison: two studies compared MTB + WJ to MTB + floss on orthodontic patients [27,32].
- Group C comparison: nine studies compared MTB + WJ to MTB alone in regular patients [28,29,30,33,34,42,44,45,46].
- Group D comparison: five studies compared MTB + WJ to MTB alone in orthodontic patients [31,32,35,41,47].

**Table 1:** Overview of the studies included.

Author/Year	Country	Design	Control Groups	Test Groups	Groups Included in This Review	Other Groups not Included in This Review	Total Participants ^§^/Drop Out %	Age Range (Mean)	Male/ Female %	Timing of Outcome Measurements	Outcomes Measured
**(A) Studies compared waterjet to manual floss on regular patients**											
Barnes/2005 [25]	USA	RCT	MB + MF (*n* = 35)	MB + WJ (*n* = 35)	2/3	SB + WJ	70/10%	19–70	NA	2 weeks 4 weeks	PI, GI, BoP
Rosema/ 2011 [26]	Netherlands	RCT	MB + MF (*n* = 36)	MB + WJ (*n* = 36) (standard tip)	2/3	MB + WJ (prototype tip)	72/2.8%	18–36 (21.8)	29/71%	2 weeks 4 weeks	PI, BoP
Akram/2015 ^&^ [42]	Iraq	RCT	MB alone (*n* = 15) MB + MF (*n* = 15)	MB + WJ (*n* = 15)	3/3	NA	45/0%	25–50	NA	3 weeks 6 weeks	PI, GI, BoP
Sasikumar/2016 [43]	India	RCT	MB + MF	MB + WJ	2/2	NA	70/8.6%	18–38	44/56%	2 weeks 4 weeks	PI, GI, BoP
**(B) Studies compared waterjet to manual floss on patients with a fixed orthodontic appliance**											
Sharma/2008 [32]	Canada	RCT	MB alone (*n* = 35) MB + MF (*n* = 35)	MB + WJ (*n* = 36)	3/3	NA	106/0.9%	11–17 (13.6)	55.7/44.3%	2 weeks 4 weeks	PI, BoP
Bruce/2013 [27]	USA	Split mouth	MB + MF (*n* = 40)	MB + WJ (*n* = 40)	2/2	NA	40/0%	13–20 (15.5)	52.5/47.5%	4 weeks	PI, GI, BoP
**(C) Studies compared waterjet to brushing alone on regular patients**											
Flemmig/1990 [45]	USA	RCT	MB alone (*n*= 55)	MB + WJ (*n*= 54)	2/4	- CHX irrigation - CHX rinse	109/ 11% at 3 M, 15.6% at 6 M	(36.6)	NA	3 months 6 months	PI, GI, BoP
Jolkovsky/1990 [28]	USA	RCT	MB alone (*n* = 15)	MB + WJ (*n* = 15)	2/4	Prof. irr: - 0.12% CHX - 0.04% CHX	30/3%	22–75 (56)	80/20%	3 months	PI, GI
Chaves/1994 [29]	USA	RCT	MB alone (*n* = 31)	MB + WJ (*n* = 32)	2/4	- 0.125 CHX rinse - 0.04% CHX irr	63/6.3%	19–62	32/68%	3 months 6 months	PI, GI, BoP
Newman/1994 [30]	Spain, Italy, Germany, France	RCT	MB alone (*n* = 59)	MB + WJ (*n* = 58)	2/3	zinc sulfate irr	117/1.7%	18–75	NA	6 months	PI, GI, BoP
Flemmig/1995 [44]	Germany	RCT	MB alone (*n* = 20)	MB + WJ (*n* = 20)	2/3	0.3% ASA irr	40/7.5%	19–75	NA	6 months	PI, GI, BoP
Cutler/2000 [33]	USA	RCT	MB alone (*n* = 20)	MB + WJ (*n* = 20)	2/3	No oral hygiene	40/0%	(Test 40.4) (Control 44)	45/55%	2 weeks	PI, GI, BoP
Ernst/2004 * [46]	Germany	RCT	MB alone (*n* = 15)	MB + WJ (Subgingival tip) (*n* = 15)	2/3	Herbal mouth rinse	30/NA	46.4	NA	4 weeks 8 weeks 3 months	PI, GI, BoP
Akram/2015 [42]	Iraq	RCT	MB alone (*n* = 15) MB + MF (*n* = 15)	MB + WJ (*n* = 15)	3/3	NA	45/0%	25–50	NA	3 weeks 6 weeks	PI, GI, BoP
Goyal/2018 [34]	Canada	RCT	MB alone (*n* = 36)	MB + WJ (*n* = 36)	2/2	NA	72/0%	25–70 (48.4)	22/78%	2 weeks 4 weeks	PI, GI, BoP
**(D) Studies compared waterjet to brushing alone on patients with a fixed orthodontic appliance**											
Jackson/1991 [31]	USA	Cross over	MB only (*n* = 20)	MB + WJ (*n* = 20)	2/4	PB only PB + WJ	20/0%	NA	40/6%	4 weeks	PI, GI
Burch/1994 [35]	USA	RCT	MB only (*n* = 15)	MB + WJ (*n* = 16)	2/3	PB + WJ	31/0%	21–48	NA	4 weeks 8 weeks	PI, GI, BoP
Sharma/2008 ^&^ [32]	Canada	RCT	MB alone (*n* = 35) MB + MF (*n* = 35)	MB + WJ (*n* = 36)	3/3	NA	106/0.9%	11–17 (13.6)	55.7/ 44.3%	2 weeks 4 weeks	PI, BoP
Patel/2015 [47]	India	RCT	MB only (*n* = 15)	MB + WJ (*n* = 15)	2/4	PB only PB + WJ	30/0%	21–22	NA	4 weeks 8 weeks	PI, GI
Mazzoleni/2019 * [41]	Italy	Split mouth	MB only (*n* = 20)	MB + WJ (*n* = 20)	2/2	NA	20/NA	13–32	50/5%	1 month 3 months 6 months	PI, GI

MB: manual brush, WJ: water jet, MF: manual floss, SB: sonic brush, PI: plaque index, GI: gingival index, BoP: bleeding on probing, CHX: chlorohexidine, ASA: acetylsalicylic acid, irr: irrigation, Prof. irr: professional irrigation, §: total participants of the included groups only, *: patients in all groups were encouraged to floss, and ^&^: studies repeated twice because they have more than one comparison included in this review.

### 3.4. Outcome Measurement Indices

Most studies used the PI by Silness and Loe (See Supplementary Table S1) to assess the plaque score, the percentage of bleeding sites to assess bleeding, and the GI by Löe and Silness (See Supplementary Table S1, which lists all the indices used in the included studies).

### 3.5. Outcome Measurement Timepoints

As the outcomes in the studies were measured at different time points, a subgroup analysis was conducted to address the short-, intermediate-, and long-term results. Short-term results, measured at 2–3 weeks, were obtained from seven studies [25,26,32,33,34,42,43]. Intermediate results, measured at 4–6 weeks, were retrieved from 12 studies [25,26,27,31,32,34,35,41,42,43,46,47]. Long-term results, which were longer than 6 weeks, were from six studies [28,29,35,41,46,47].

### 3.6. Risk of Bias

**Figure 2:**
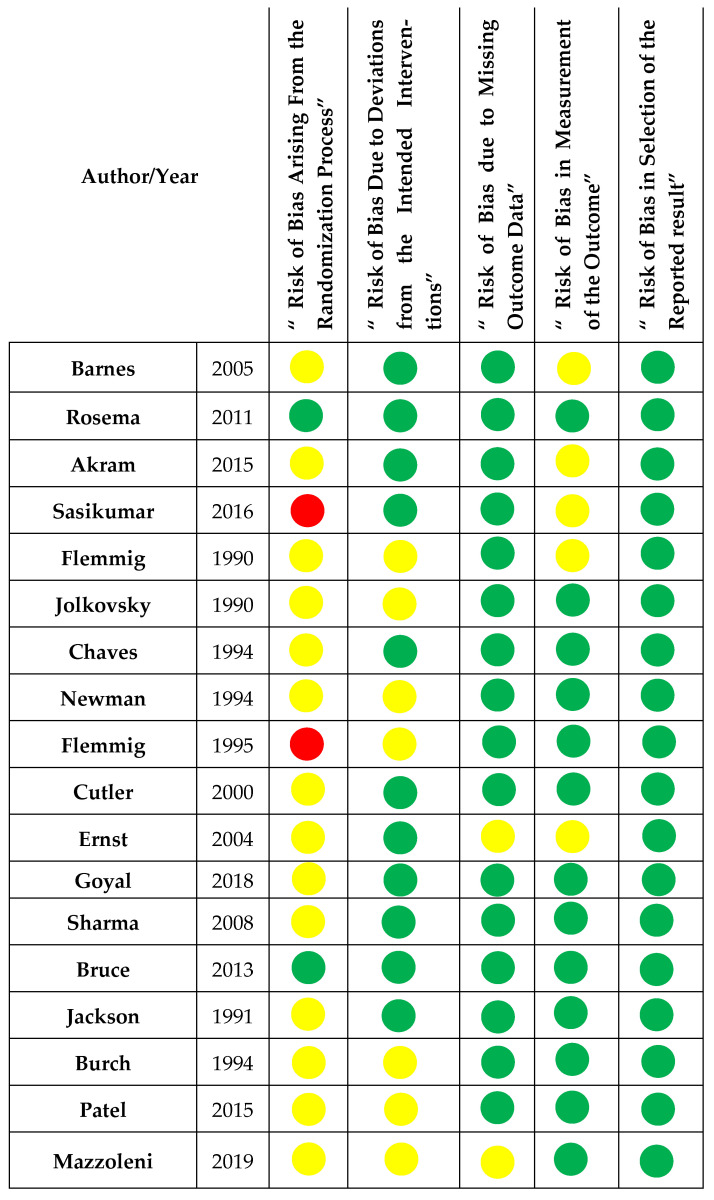
Risk of bias according to Cochrane risk of bias 2 (ROB 2) [25,26,27,28,29,30,31,32,33,34,35,41,42,43,44,45,46,47]. green: low risk, yellow: some concerns, and red: high risk.

A summary of the ROB assessment is shown in Figure 2. Our thorough evaluation of bias in the randomization process led us to identify two studies with a low risk of bias, owing to detailed descriptions of the concealment process [26,27]. Conversely, a high risk of bias was assigned to two studies [43,44] where a significant baseline imbalance between the groups was observed. The rest of the studies were given the judgment of “some concern” as the concealment process was not mentioned, but there was no baseline imbalance between the groups.

Bias due to deviation from the intended intervention was low in most studies and of some concern in seven studies. Although blinding of participants was not applicable in all trials, a deviation that arose because of the trial context was not expected in eleven studies [25,26,27,29,31,32,33,34,42,43,46]. In contrast, deviation was expected in seven trials but was not expected to affect study outcomes [28,30,35,41,44,45,47].

The ROB due to missing outcome data was low in sixteen studies. Seven of them had all participants complete the trial and there were no missing data [27,31,33,34,35,42,47]. Three studies had less than 5% dropout [28,30,32]. Six studies had up to 10% dropout, but this appeared random across the groups and possibly did not affect the outcome [25,26,29,43,44,45]. Only two studies were of some concern because there was no information about dropouts [41,46]. However, it is unlikely that missing data would be related to the outcome.

Thirteen trials showed a low risk from outcome measurement due to the use of well-known indices, and clear explanations of assessor blinding [26,27,28,29,30,31,32,33,34,35,41,44,47]. Some concerns were assigned to five studies since the only information mentioned regarding assessors blinding was the phrase “single-blinded”, with no further details [25,42,43,45,46]. Finally, regarding the selection of the reported results, all the studies were at a low ROB because all outcomes mentioned in their methods were reported in the results.

### 3.7. Descriptive Analysis of the Effect of WJs

#### 3.7.1. Group A Comparison: WJ + MTB vs. Flossing + MTB (Regular Patients, Four Studies)

All studies concluded that there was no significant difference between using a WJ and flossing in terms of plaque reduction at 2–3 weeks and 4–6 weeks. However, three out of four studies reported significant improvements in BI and GI in favor of the WJ group at all follow-up periods.

#### 3.7.2. Group B Comparison: WJ + MTB vs. Flossing + MTB (Orthodontic Patients, Two Studies)

One study showed significant improvement in PI and BI for WJ users at 2 and 4 weeks, while the other found no significant differences between WJ use and flossing.

#### 3.7.3. Group C Comparison: WJ + MTB vs. MTB Alone (Regular Patients, Nine Studies)

WJ users showed significant improvement in PI at 2 and 4 weeks (two studies), but not at 3–6 months (six studies). Regarding bleeding, significant improvements for WJ users were observed at 2–4 weeks, while long-term results were inconsistent (five studies). For GI, WJ users showed significant improvement at 2–4 weeks (two studies) and 3–6 months (four studies).

#### 3.7.4. Group D Comparison: WJ + MTB vs. MBT Alone (Orthodontic Patients, Five Studies)

Three studies showed equal plaque reduction at 4–8 weeks, while two indicated superior results for the WJ users at 2–8 weeks. For BI, which was assessed in two studies, both favored WJ users at 2, 4, and 8 weeks. Regarding GI, one study found the results for WJ users to be superior at 4–8 weeks, but three others found no significant differences between the groups from 4 weeks to 6 months (see Supplementary Table S2).

### 3.8. Quantitative Analysis of the Effect WJ on BI, PI, and GI

#### 3.8.1. Bleeding Index

- Group A comparison: WJ + MTB versus floss + MTB in regular patients (Table 2: Grade summary of finding One)

**Figure 3:**
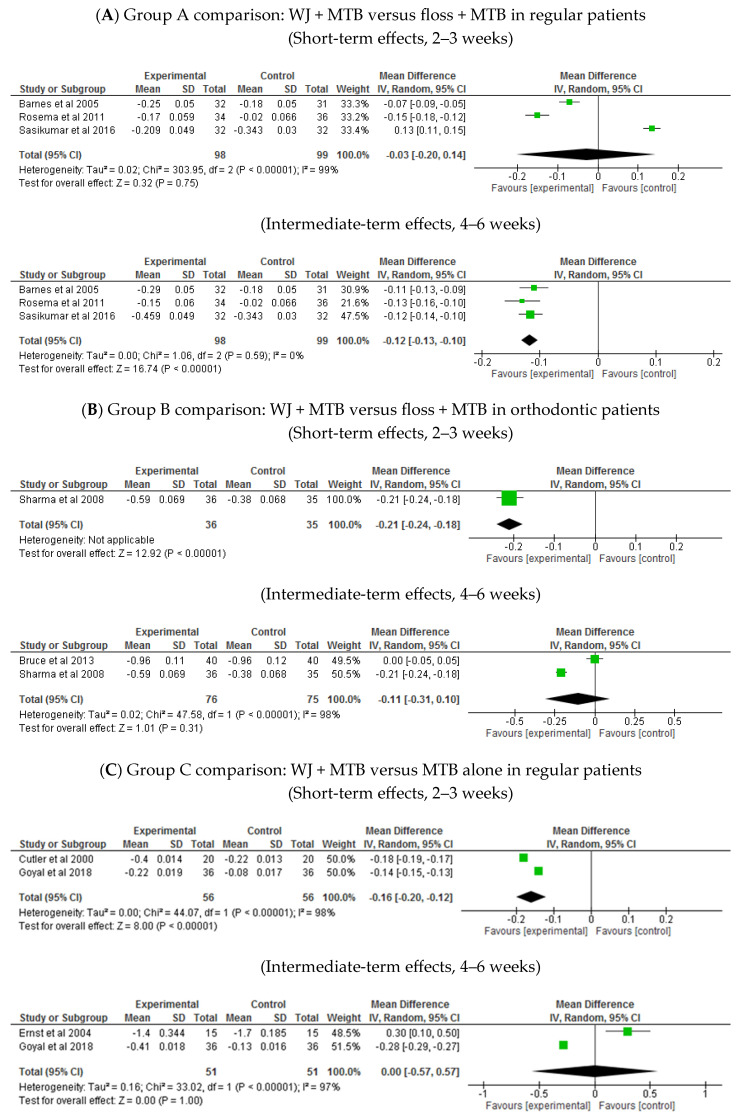

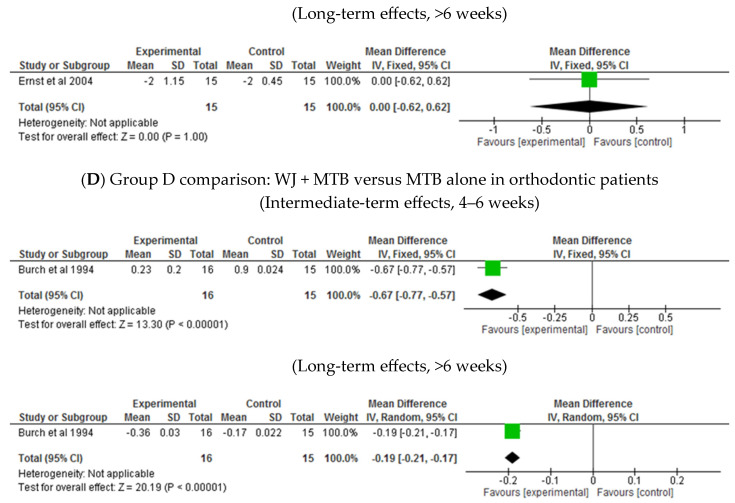
Bleeding index [25,26,27,32,33,34,35,43,46].

At 4–6 weeks, moderate-certainty evidence indicated that bleeding reduction was slightly higher in the WJ group compared to the flossing group (MD −0.12, 95% CI: −0.13 to −0.10, *p* < 0.00001; three trials; Figure 3A). However, there was no significant difference between the groups at 2–3 weeks (MD −0.03, 95% CI: −0.2 to 0.14, *p* = 0.75; 3 trials; Figure 3A).

- Group B comparison: WJ + MTB versus floss + MTB in orthodontic patients (Table 3: Grade summary of finding Two)

The effect of WJ use on bleeding reduction was slightly superior to that of flossing in orthodontic patients at 2 weeks (MD −0.21, 95% CI: −0.24 to −0.18, *p* < 0.00001; one trial; Figure 3B). However, the difference between WJ use and flossing was not significant at 4 weeks (MD: −0.11, 95% CI: −0.31 to 0.10, *p* = 0.31; two trials; Figure 3B; moderate-certainty evidence).

- Group C comparison: WJ + MTB versus MTB alone in regular patients (Table 4: Grade summary of finding Three)

Moderate-certainty evidence showed that bleeding reduction was slightly greater in the WJ group at 2 weeks (MD −0.16, 95% CI: −0.02 to −0.12, *p* < 0.00001; two trials; Figure 3C). However, the effect was similar in both groups at 1 month (MD 0.00, 95% CI: −0.57 to −0.57, *p* = 1; two trials; Figure 3C) and 2 months.

- Group D comparison: WJ + MTB versus MTB alone in orthodontic patients (Table 5: Grade summary of finding Four)

Only one trial in this comparison evaluated BI. It showed superior effect for WJ use compared to MTB alone at 1 month (MD −0.67, 95% CI: −0.77 to −0.57, *p* < 0.00001; one trial; Figure 3D) and 2 months (MD −0.19, 95% CI: −0.21 to −0.17, *p* < 0.00001; one trial; Figure 3D).

#### 3.8.2. Plaque Index

- Group A comparison: WJ + MTB versus floss + MTB in regular patients (Table 2: Grade summary of finding One)

**Figure 4:**
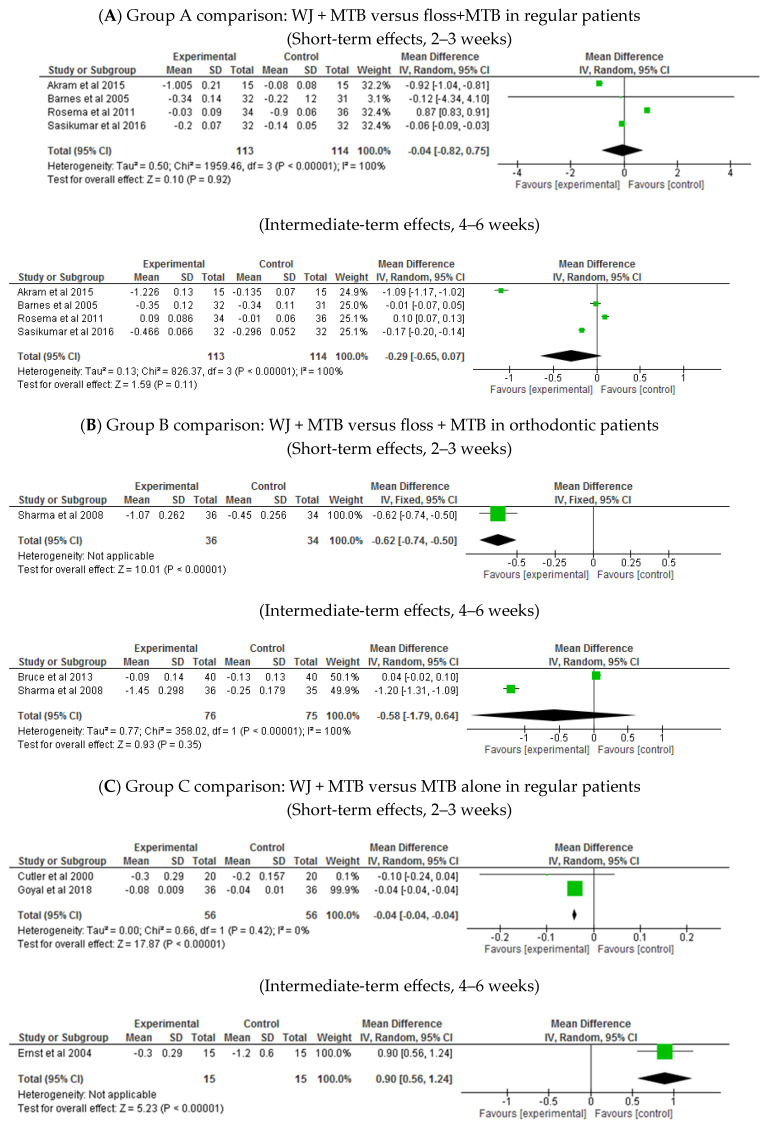

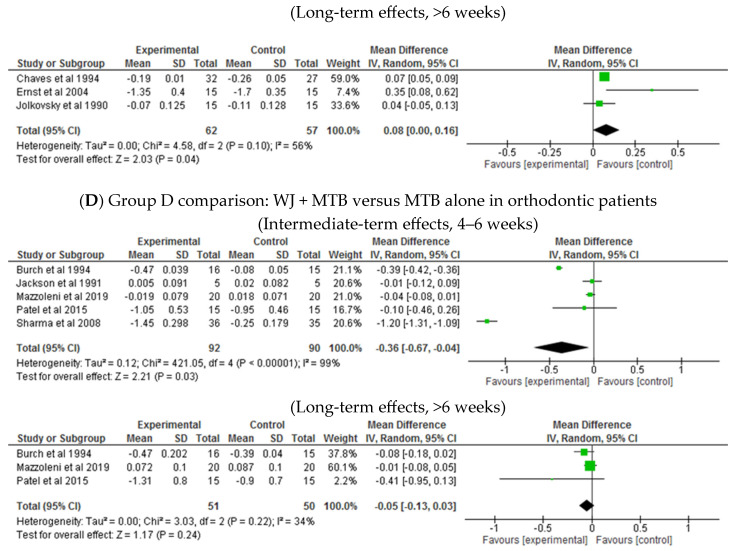
Plaque index [25,26,27,28,29,31,32,33,34,35,41,42,43,46,47].

At 2–3 weeks, there was no significant difference on plaque reduction between the WJ and floss groups (MD −0.04, 95% CI: −0.82 to −0.75, *p* = 0.92; four trials; Figure 4A). A similar finding was also observed at 4–6 weeks (MD −0.29, 95% CI: −0.65 to 0.07, *p* = 0.1; four trials; Figure 4A; low-certainty evidence).

- Group B comparison: WJ + MTB versus floss + MTB in orthodontic patients (Table 3: Grade summary of finding Two)

At 2 weeks, WJ showed slightly better plaque reduction than flossing (MD −0.62, 95% CI: −0.74 to −0.5, *p* = 0.00001; one trial; Figure 4B). However, at 4 weeks, there was no significant difference between the groups (MD −0.58, 95% CI: −1.79 to 0.64, *p* = 0.35; two trials; Figure 4B; low-certainty evidence).

- Group C comparison: WJ + MTB versus MTB alone in regular patients (Table 4: Grade summary of finding Three)

At 2 weeks, WJ provided slightly greater plaque reduction than MTB alone (MD −0.04, 95% CI: −0.04 to −0.04, *p* < 0.00001; two trials; Figure 4C). However, MTB-alone showed superior results at 4 weeks (0.9, 95% CI: 0.56 to 1.24, *p* < 0.00001; one trial; Figure 4C) and 2–3 months (0.08, 95% CI: 0.00 to 0.16, *p* = 0.04; three trials; Figure 4C).

- Group D comparison: WJ + MTB versus MTB alone in orthodontic patients (Table 5: Grade summary of finding Four)

At 4 weeks, WJ was superior to MTB alone in reducing plaque (MD −0.36, 95% CI: −0.67 to −0.04, *p* = 0.03; five trials; Figure 4D). However, at 2–3 months, the difference between the two groups was not statistically significant (MD −0.05, 95% CI: −0.13 to 0.03, *p* = 0.24; three trials; Figure 4D).

#### 3.8.3. Gingival Index

- Group A comparison: WJ + MTB versus floss + MTB in regular patients (Table 2: Grade summary of finding One)

**Figure 5:**
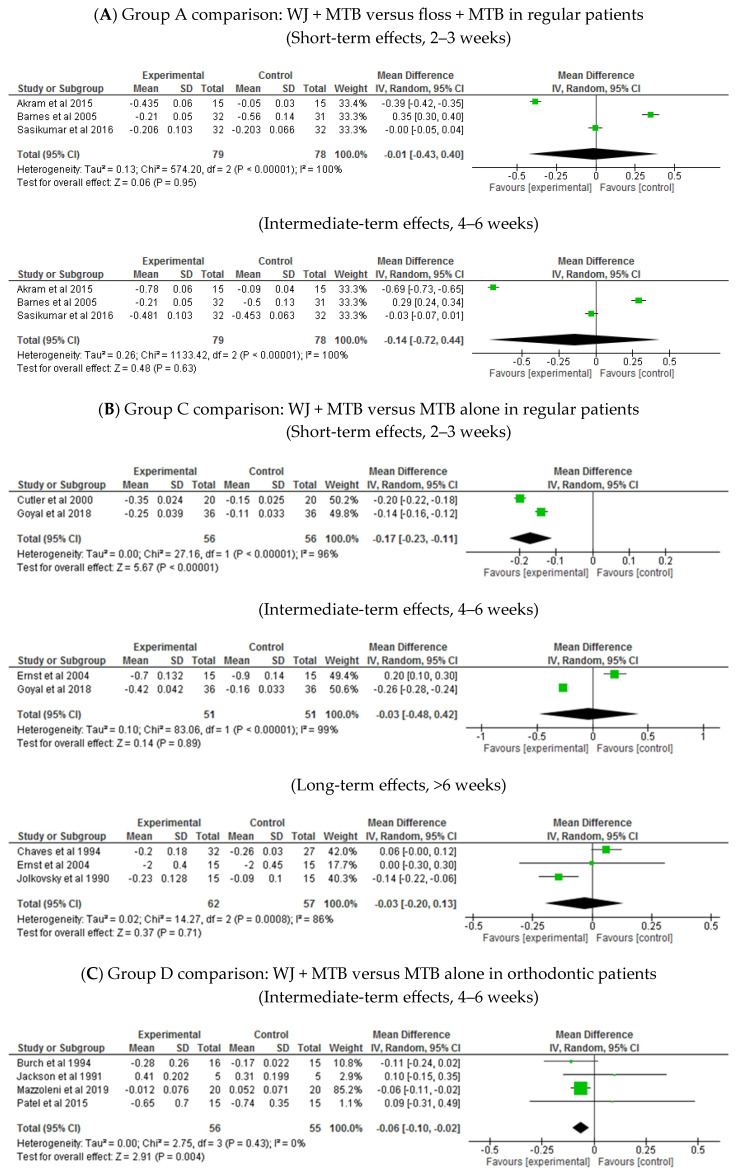

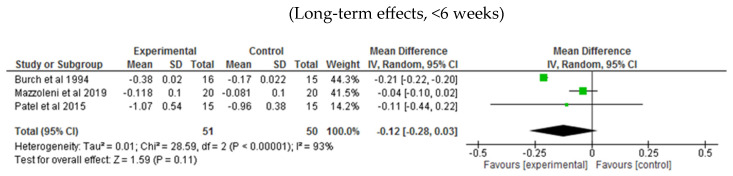
Gingival index [25,28,29,31,33,34,35,41,42,43,46,47].

The effect of WJ use on the gingival index was similar to that for floss use in regular patients at both 2–3 weeks (MD −0.01, 95% CI: −0.43 to 0.4, *p* = 0.95; three trials; Figure 5A) and at 4–6 weeks (MD −0.14, 95% CI: −0.72 to 0.44, *p* = 0.63; three trials; Figure 5A).

- Group C comparison: WJ + MTB versus MTB alone in regular patients (Table 4: Grade summary of finding Three)

At 2 weeks, WJ use had a slightly better effect on the GI than MTB alone (MD −0.17, 95% CI: −0.23 to −0.11, *p* < 0.00001; two trials; Figure 5B). However, this superior effect was not observed at 4 weeks (MD −0.03, 95% CI: −0.48 to 0.42, *p* = 0.89; two trials; Figure 5B) or at 3 months (MD −0.03, 95% CI: −0.2 to 0.13, *p* = 0.71; three trials; Figure 5B).

- Group D comparison: WJ + MTB versus MTB alone in orthodontic patients (Table 5: Grade summary of finding Four)

Compared to MTB alone, WJ use showed minimal improvement in GI at 4 weeks (MD −0.06, 95% CI: −0.1 to −0.02, *p* = 0.004; five trials; Figure 5C). AT 2–3 months, the difference between WJ use and MTB alone was not statistically significant (MD −0.12, 95% CI: −0.28 to 0.03, *p* = 0.11; three trials; Figure 5C).

**Table 2:** GRADE Summary of Findings One. Group A comparison: Waterjet plus manual toothbrushing compared to toothbrushing plus flossing for regular patients.

Patient or population: regular patients. Setting: daily oral care Intervention: waterjet Comparison: manual floss						
Outcomes	Anticipated absolute effects * (95% CI)		Relative effect (95% CI)	№ of participants (studies)	Certainty of the evidence (GRADE)	Comments
	**Regular floss**	**Waterjet**				
Reduction in plaque index (PI) (lower scores means less plaque) follow-up: range 4 to 6 weeks	The mean reduction in PI was **−0.2**	MD **0.29 lower** (0.65 lower to 0.07 higher)	-	227 (4 RCTs)	⨁⨁◯◯ Low ^a,b^	In regular patients, results showed equal benefits from waterjet and regular flossing in improving PI at 4–6 weeks. A similar effect was observed also in shorter-term (MD −0.04 [−0.82, 0.75], *p* = 0.92, 4 trials)
Reduction in bleeding index (BI) (lower scores means less bleeding) follow-up: range 4 to 6 weeks	The mean reduction in BI was **−0.18**	MD **0.12 lower** (0.13 lower to 0.1 lower)	-	197 (3 RCTs)	⨁⨁⨁◯ Moderate ^a^	In regular patients, waterjet likely has a slightly better effect than regular floss in improving BI at four weeks and longer; shorter-term results showed no superior benefit for water jet over flossing (MD −0.03 [−0.20, 0.14], *p* < 0.75, 3 trials)
Reduction in gingival index (GI) (lower scores mean lower degree of gingivitis) follow-up: range 4 to 6 weeks	The mean reduction in GI was **−0.35**	MD **0.14 lower** (0.72 lower to 0.44 higher)	-	157 (3 RCTs)	⨁◯◯◯ Very low ^a,c^	In regular patients, waterjet may result in no difference in improving GI over regular flossing; shorter-term results also have no benefit over flossing (MD −0.01 [−0.03, 0.40], *p* = 0.95, 3 trials)
* **The risk in the intervention group** (and its 95% confidence interval) is based on the assumed risk in the comparison group and the **relative effect** of the intervention (and its 95% CI). **CI:** confidence interval; **MD:** mean difference						
**GRADE Working Group grades of evidence** **High certainty:** we are very confident that the true effect lies close to that of the estimate of the effect. **Moderate certainty:** we are moderately confident in the effect estimate: the true effect is likely to be close to the estimate of the effect, but there is a possibility that it is substantially different. **Low certainty:** our confidence in the effect estimate is limited: the true effect may be substantially different from the estimate of the effect. **Very low certainty:** we have very little confidence in the effect estimate: the true effect is likely to be substantially different from the estimate of effect.						

^a^ One Study had a high risk of bias arising from the randomization process and 2 studies had unclear risk of bias. ^b^ There is visual inconsistency in the plot chart, and the statistical test shows heterogeneity. ^c^ Statistical analysis shows substantial heterogeneity.

**Table 3:** GRADE Summary of Findings Two. Group B comparison: Waterjet plus manual toothbrushing compared to toothbrushing plus flossing for orthodontic patients.

Patient or population: patients with fixed orthodontic appliance Setting: daily oral care Intervention: water jet Comparison: regular floss						
Outcomes	Anticipated absolute effects * (95% CI)		Relative effect (95% CI)	№ of participants (studies)	Certainty of the evidence (GRADE)	Comments
	**Regular floss**	**Water jet**				
Reduction in plaque index (PI) (lower score means less plaque) follow-up: range 4 to 6 weeks	The mean reduction in PI was **−0.19**	MD **0.58 lower** (1.79 lower to 0.64 higher)	-	151 (2 RCTs)	⨁⨁◯◯ Low ^a^	In orthodontic patients, no difference was observed between water jet and manual flossing in improving PI at 4–6 weeks. Shorter-term showed slightly higher benefit for water jet (MD −0.62 [−0.74, −0.50], *p* < 0.00001, 1 trial)
Reduction in bleeding index (BI) (lower score means less bleeding) follow-up: range 4 to 6 weeks	The mean reduction in BI was **−0.67**	MD **0.11 lower** (0.31 lower to 0.1 higher)	-	151 (2 RCTs)	⨁⨁⨁◯ Moderate ^b^	In orthodontic patients, water jet probably had similar effect on bleeding when compared to regular floss at 4–6 weeks; shorter-term showed slightly higher benefit for water jet over floss (MD −0.21 [−0.24, −0.18], *p* < 0.00001, 1 trial)
Reduction in gingival index (GI) not measured	No included studies comparing water jet to regular floss in orthodontic patients reported gingival index.					
* **The risk in the intervention group** (and its 95% confidence interval) is based on the assumed risk in the comparison group and the **relative effect** of the intervention (and its 95% CI). **CI:** confidence interval; **MD:** mean difference						
**GRADE Working Group grades of evidence** **High certainty:** we are very confident that the true effect lies close to that of the estimate of the effect. **Moderate certainty:** we are moderately confident in the effect estimate: the true effect is likely to be close to the estimate of the effect, but there is a possibility that it is substantially different. **Low certainty:** our confidence in the effect estimate is limited: the true effect may be substantially different from the estimate of the effect. **Very low certainty:** we have very little confidence in the effect estimate: the true effect is likely to be substantially different from the estimate of effect.						

^a^ There is visual inconsistency in the plot chart, and statistical analysis also shows substantial heterogeneity. ^b^ There is visual inconsistency in the plot chart, and statistical analysis also shows heterogeneity.

**Table 4:** GRADE Summary of Findings Three. Group C comparison: Waterjet plus manual toothbrushing compared to toothbrushing alone for regular patients.

**Patient or population:** regular patients **Setting:** daily oral care **Intervention:** waterjet plus toothbrushing **Comparison:** toothbrushing alone						
Outcomes	Anticipated absolute effects * (95% CI)		Relative effect (95% CI)	№ of participants (studies)	Certainty of the evidence (GRADE)	Comments
	**Toothbrushing** **alone**	**Waterjet plus** **toothbrushing**				
Reduction in plaque index (PI) (lower score means less plaque) follow-up: range 2 to 3 weeks	The mean reduction in PI was **−0.12**	MD **0.04 lower** (0.04 lower to 0.04 lower)	-	112 (2 RCTs)	⨁⨁◯◯ Low ^a^	In regular patients, waterjet plus toothbrushing may result in a very slight difference in improving PI than brushing alone in the short term of 3 weeks. Longer-term evidence (4–6 weeks) showed higher benefit for brushing alone (MD 0.90 [0.56, 1.24], *p* < 0.00001, 1 trial) as well as three-months data (MD 0.08 [0.00, 0.16], *p* = 0.04, 3 trial)
Reduction in bleeding index (BI) (lower score means less bleeding) follow-up: range 2 to 3 weeks	The mean reduction in BI was **−0.15**	MD **0.16 lower** (0.2 lower to 0.12 lower)	-	112 (2 RCTs)	⨁⨁⨁◯ Moderate ^b^	In regular patients, waterjet plus toothbrushing may improve BI slightly more than brushing alone at 2–3 weeks; longer-term results (1 and 2 months) showed an equal effect of both (MD 0.00 [−0.6, −0.6], *p* = 1.00, 3 trials)
Reduction in gingival index (GI) (lower score means lower degree of gingivitis) follow-up: range 2 to 3 weeks	The mean reduction in GI was **−0.13**	MD **0.17 lower** (0.23 lower to 0.11 lower)	-	112( 2 RCTs)	⨁⨁⨁◯ Moderate ^b^	In regular patients, waterjet plus toothbrushing may improve GI slightly more than brushing alone at 2–3 weeks; longer-term results showed an equal effect of both (MD −0.03 [−0.20, 0.13], *p* = 0.71, 3 trials)
* **The risk in the intervention group** (and its 95% confidence interval) is based on the assumed risk in the comparison group and the **relative effect** of the intervention (and its 95% CI). **CI:** confidence interval; **MD:** mean difference						
**GRADE Working Group grades of evidence** **High certainty:** we are very confident that the true effect lies close to that of the estimate of the effect. **Moderate certainty:** we are moderately confident in the effect estimate: the true effect is likely to be close to the estimate of the effect, but there is a possibility that it is substantially different. **Low certainty:** our confidence in the effect estimate is limited: the true effect may be substantially different from the estimate of the effect. **Very low certainty:** we have very little confidence in the effect estimate: the true effect is likely to be substantially different from the estimate of effect.						

^a^ Downgraded two levels as studies show substantially imprecise estimate. ^b^ There is visual inconsistency in the plot chart, and statistical analysis also shows heterogeneity.

**Table 5:** GRADE Summary of Findings Four. Group D comparison: Waterjet plus manual toothbrushing compared to toothbrushing alone for orthodontic patients.

Patient or population: patients with fixed orthodontic appliance Setting: daily oral care Intervention: waterjet plus toothbrushing Comparison: toothbrushing alone						
Outcomes	Anticipated absolute effects * (95% CI)		Relative effect (95% CI)	№ of participants (studies)	Certainty of the evidence (GRADE)	Comments
	**Toothbrushing** **alone**	**Waterjet plus** **toothbrushing**				
Reduction in plaque index (PI) (lower score means less plaque) follow-up: range 4 to 6 weeks	The mean reduction in PI was **−0.25**	MD **0.36 lower** (0.67 lower to 0.04 lower)	-	182 (5 RCTs)	⨁◯◯◯ Very low ^a,b^	In orthodontic patients, waterjet may result in a slight additional benefit over regular flossing in improving PI at 4–6 weeks; longer-term results showed no difference over flossing (MD −0.05 [−0.13, 0.03], *p* = 0.24, 3 trials)
Reduction in bleeding index (BI) (lower score means less bleeding) follow-up: range 4 to 6 weeks	The mean reduction in BI was **0.9**	MD **0.67 lower** (0.77 lower to 0.57 lower)	-	31 (1 RCT)	⨁⨁⨁◯ Moderate ^c^	In orthodontic patients, waterjet may result in slight additional benefit over regular flossing in improving BI at 4 weeks; also, at 2 months (MD −0.19 [−0.21, −0.17], *p* < 0.00001, 1 trial)
Reduction in gingival index (GI) (lower score means lower degree of gingivitis) follow-up: range 4 to 6 weeks	The mean reduction in GI was **−0.14**	MD **0.06 lower** (0.1 lower to 0.02 lower)	-	111 (4 RCTs)	⨁◯◯◯ Very low ^a,b^	In orthodontic patients, waterjet may result in little to no difference in improving GI over brushing alone at 4–6 weeks; in the longer-term, results showed no difference between the two groups (MD −0.12 [−0.28, 0.03], *p* = 0.11, 3 trials)
* **The risk in the intervention group** (and its 95% confidence interval) is based on the assumed risk in the comparison group and the **relative effect** of the intervention (and its 95% CI). **CI:** confidence interval; **MD:** mean difference						
**GRADE Working Group grades of evidence** **High certainty:** we are very confident that the true effect lies close to that of the estimate of the effect. **Moderate certainty:** we are moderately confident in the effect estimate: the true effect is likely to be close to the estimate of the effect, but there is a possibility that it is substantially different. **Low certainty:** our confidence in the effect estimate is limited: the true effect may be substantially different from the estimate of the effect. **Very low certainty:** we have very little confidence in the effect estimate: the true effect is likely to be substantially different from the estimate of effect.						

^a^ There is visual inconsistency in the plot chart and statistical tests show substantial heterogeneity. ^b^ Studies show substantially imprecise estimate. ^c^ Studies show moderate imprecise estimate.

## 4. Discussion

This comprehensive review investigated the benefits of dental WJ use in daily home-based dental care to enhance plaque removal and improve gingival health in regular and orthodontic populations. Comparisons were made between four groups: WJ + MTB versus MTB + flossing in regular patients, WJ + MTB versus MTB + flossing in orthodontic patients, WJ + MTB versus MTB alone in regular patients, and WJ + MTB versus MTB alone in orthodontic patients. To reduce heterogeneity, subgroup analyses were conducted at three follow-up periods: short (2–3 weeks), intermediate (4–6 weeks), and long (>6 weeks).

Regarding plaque, in regular patients, low-certainty evidence showed that, in the short and intermediate terms, WJ use did not improve PI more than flossing, and the results were inconsistent when compared to MTB alone. In orthodontic patients, WJ use appeared more beneficial when compared to flossing. Low-certainty evidence showed a slightly better short-term outcome for the WJ users. When compared to MTB alone, very low-certainty evidence showed a better intermediate-term outcome, but no significant long-term advantage, for the WJ users. Given that orthodontic appliances make oral hygiene challenging, WJs could serve as a valuable adjunct to improve plaque removal and overall oral care compared to flossing or brushing alone.

Regarding the bleeding index, moderate-certainty evidence suggested that WJ use slightly improved BI, more than flossing, in regular patients in the intermediate, but not the short, term. Compared to MTB alone, WJ use showed only a better short-term effect. The impact of WJ use was more pronounced among orthodontic patients, where it demonstrated a slightly better short-term impact than flossing and a higher effect at all timepoints compared to MTB alone. Since using a WJ is easier than flossing, it might reduce the difficulty of maintaining good oral hygiene and increase patient compliance, positively affecting gingival health.

For the gingival index, very low-certainty evidence showed that WJ use and flossing had similar effects for regular patients. In contrast, compared to brushing alone, WJ use showed a slightly higher short-term benefit for regular patients (moderate certainty) and an intermediate-term benefit for orthodontic patients (very low certainty).

Husseini et al. (2008) systematically reviewed the benefits of oral irrigation as an adjunct to brushing, compared to brushing alone or regular oral hygiene. Two studies revealed significant improvements in PI in favor of the irrigation group, whereas three demonstrated better outcomes in bleeding and gingival indices. Thus, the results showed a promising trend in favor of oral irrigation over regular oral hygiene for potentially enhancing gingival health. This aligns with our finding that WJ use provided a slightly greater benefit in terms of bleeding and gingival indices at certain time points [36].

Kotsakis et al. (2018) conducted a network meta-analysis ranking ten interproximal oral hygiene tools from 22 trials based on their benefits in terms of reducing gingival inflammation. A WJ was ranked as the second-best tool for improving GI and BI, though it did not offer additional benefits in terms of PI reduction compared to the control (brushing only). Our findings align with this, showing a slight short-term advantage of WJ use over MTB for BI and GI, and a longer-term improvement in BI compared to flossing [18]. Unlike Kotsakis et al., our study included more articles that compared WJ use and MTB alone, and excluded papers that used powered brushes [48], or powered flossers that differed from WJs or irrigation devices [49], which were included in Kotsakis’ review. Additionally, Kotsakis et al. did not subgroup the results by time points, whereas our study accounted for short-, intermediate-, and long-term effects.

Worthington et al. (2019) published a comprehensive meta-analysis comparing the effectiveness of different interproximal tools to brushing alone and to each other. They found that, at 1 month only, WJ use may reduce GI more than brushing alone and flossing but led to no significant differences in PI, BI, or GI [19]. Our data align with this, showing a slightly improved effect of WJ use on GI compared to MTB alone but not against flossing at 2 weeks. Similarly, our study did not find WJs to be superior for plaque reduction. However, we found a positive impact of WJ use on bleeding at 2 weeks compared to MBT and at 4 weeks compared to flossing. These variations may be attributed to differences in the studies included in the analysis. Unlike Worthington et al., we included additional studies comparing WJs to floss [42,43] and WJ use to MTB [28,29,33,34,46]. These studies were selected based on their relevance to the research questions and inclusion criteria. Moreover, we restricted our selection to studies published from 1990 onward and excluded papers that involved powered brushes rather than manual brushes [48].

A recent systematic review and meta-analysis by Mohapatra et al. (2023) compared WJ use to flossing and found that PI reduction was significantly higher with WJ use. This differs from our results, which showed no difference in PI reduction between WJ use and flossing. This discrepancy could be explained by the studies included in their analysis: four of the six studies in Mohapatra’s analysis lacked a follow-up period, and assessments of outcome were conducted just before and after cleaning [50]. In contrast, our study included only studies with a follow-up period of at least 2 weeks.

Another recent meta-analysis by Almoharib et al. (2023) showed results comparable to ours, where WJ use was more effective in reducing PI and bleeding than MTB alone in an orthodontic population [51].

## 5. Clinical Implications and Recommendation

Our findings suggest that WJ use may be beneficial as an adjunct to oral hygiene, especially for orthodontic patients who face challenges with the daily dental care to maintain adequate plaque control. Given its ease of use and slight advantages in terms of BI and GI improvement, a WJ can be recommended for patients who find flossing difficult or ineffective. Meanwhile, it should be emphasized to patients that WJs neither replace brushing nor compensate for poor-quality brushing. While employing a WJ as an additional tool, patients should be encouraged to enhance their brushing methods.

Dental care providers may find that suggesting a WJ as an interproximal cleaning solution is particularly helpful for individuals with orthodontic brackets, fixed prostheses, or dexterity challenges, or those with a history of poor compliance with traditional flossing.

## 6. Study Limitations

Our findings may be limited by the number and quality of the included studies, as well as variations in participant criteria. Additionally, we did not analyze outcomes related to periodontitis, such as probing depth, since only a few studies included this parameter. Furthermore, the clinical significance of our results should be interpreted with caution, as the mean intergroup difference might be minimal to be clinically meaningful, and there is no established cutoff for clinically significant differences in plaque, bleeding, or gingival indices.

Future research should be conducted over the long term and adequately powered to provide more definitive conclusions. It should also consider additional outcomes relevant to patient centered care such as cost effectiveness and patients compliance.

## 7. Conclusions

Our evidence suggests a slightly higher benefit of WJ use in regular and orthodontic patients at some time points, with low to very low certainty for PI and GI and moderate certainty for bleeding. The benefits are more pronounced when WJ use is compared to MTB alone rather than flossing, and in orthodontic patients rather than in regular patients. Given its ease of use, a WJ might be more effective when flossing is challenging or when orthodontic brackets or prostheses complicate oral hygiene. We recommend adding a WJ to the daily oral hygiene routine because of its probable beneficial effects, compared to brushing, and ease of use when compared to flossing. Moreover, dental professionals should focus on motivating patients to improve the quality of brushing because interproximal aids, including WJs, are adjuncts and do not replace brushing or compensate for poor-quality brushing.

## Data Availability

The original contributions presented in this study are included in the article/Supplementary Materials. Further inquiries can be directed to the corresponding author.

## Supplementary Materials

The following supporting information can be downloaded at: https://www.mdpi.com/article/10.3390/healthcare13040396/s1, Supplementary Table S1: Outcome measurement indices: plaque, bleeding, and gingival indices used in the included studies. Supplementary Table S2: Descriptive analysis of the effect of water irrigation on the outcomes. Supplementary Box S1: List of keywords used in the search process and Supplementary Box S2: Complete search strategy on MEDLINE (PubMed).
