# Supplementary material for: Alleviation of Plaque and Gingivitis with Dental Water Jet in Regular and Orthodontic Patients: A Systematic Review and Meta-Analysis
Source: Healthcare (Basel). 2025 Feb 12;13(4):396. doi: 10.3390/healthcare13040396 (PMC11855567; doi:10.3390/healthcare13040396)
Supplement: Supplementary file 1 [file healthcare-13-00396-s001.zip › healthcare-3400383-supplementary.pdf]

Supplemantary Table S1: Outcome measurement indices: plaque, bleeding, and gingival indices used in the included studies.

Outcome	Measurement of the outcome	Studies
**Plaque index**	Proximal/Marginal Plaque Index (Benson et al., 1993)	Barnes et al., 2005.
	Turesky modification of the Quigley & Hein plaque index TQHPI (Turesky et al., 1970)	Rosema et al., 2011; Sasikumar et al., 2016; Sharma et al., 2008; Bruce et al., 2013.
	Plaque index by Silness & Löe (Silness & Löe, 1964)	Akram et al., 2015; Burch et al., 1994; Patel et al., 2015; Mazzoleni et al., 2019; Flemmig et al., 1990; Jolkovsky et al., 1990; Chaves et al., 1994; Newman et al., 1994; Flemmig et al., 1995; Cutler et al., 2000.
	Quigley & Hein plaque index (Quigley & Hein, 1962)	Ernst et al., 2004.
	Rustogi Modification of Navy Plaque Index (RMNPI) (Rustogi et al., 1992)	Goyal et al., 2018.
	Plaque index designed by the author modified from patient hygiene performance (PHP) index (Podshadley and Haley, 1968)	Jakson et al., 1991.
**Bleeding**	Carter and Barnes gingival bleeding index (Carter and Barnes, 1974)	Barnes et al., 2005.
	Bleeding on marginal probing index (BOMP) by (Van der Weijden et al., 1994)	Rosema et al., 2011; Sasikumar et al., 2016.
	Gingival bleeding index by (Saxton et al., 1993)	Sharma et al., 2008.
	Papillary bleeding index (Saxer and Muhlemann, 1975)	Bruce et al., 2013.
	Sulcus bleeding index (Muhlemann and Son, 1971)	Burch et al., 1994; Ernst et al., 2004.
	Bleeding on probing at 4 sites	Akram et al., 2015.
	Bleeding on probing at 6 sites	Flemmig et al., 1990; Chaves et al., 1994; Newman et al., 1994; Flemmig et al., 1995; Cutler et al., 2000; Goyal et al., 2018.
	Gingival index (GI) by (Löe & Silness, 1963)	Barnes et al., 2005; Akram et al., 2015; Burch et al., 1994; Mazzoleni et al., 2019; Chaves et

**Gingival index**		al., 1994; Cutler et al., 2000.
	Modified gingival index by (Löe, 1967)	Patel et al., 2015; Jakson et al., 1991; Flemmig et al., 1990; Jolkovsky et al., 1990; Newman et al., 1994; Flemmig et al., 1995; Ernst et al., 2004.
	Modified gingival index (MGI) by (Lobene et al., 1986)	Bruce et al., 2013; Goyal et al., 2018.
	Not explained and not referenced	Sasikumar et al., 2016.

Supplemantary Table S2: Descriptive analysis of the effect of water irrigation on the outcomes

Study Comparison	Author/ date	Indices impro-vement	Test (MB+WJ)		Control (MB+MF) or (MB only)		Inter-group comparison Test vs. control		Author conclusion
			BL-T1	BL- Tf	BL-T1	BL-Tf	BL-T1	BL-Tf	
**Group 1: waterjet to manual floss in gingivitis or periodontist patients**	Barnes/ 2005	PI	(2 w) S	(4 w) S	NS	S	F-S, L-NS	F-NS, L-NS	"WJ paired with a MB was statistically better at reducing bleeding and gingivitis than manual brushing and flossing, but equivalent in plaque removal"
		BOP	S	S	S	S	F-S, L-NS	F-S, L-NS	
		GI	S	S	S	S	F-S, L-NS	F-S, L-S	
	Rosema/ 2011	PI	NA	NA	NA	NA	(2 w) NS	(4 w) NS	"Within the limits of this 4-week study, the use of WJ, is significantly more effective in reducing gingival bleeding as compared to the use of MF"
		BOP					S	S	
	Akram/ 2015	PI	(3 w) S	(6 w) S	S	S	NA	NA	"WJ when combined with tooth brushing is an effective alternative to traditional MF for reducing the plaque, bleeding and gingival inflammation"
		BOP	S	S	S	S			
		GI	S	S	S	S			
	Sasikumar/ 2016	PI	(2 w) S	(4 w) S	S	S	NS	NS	"Both WJ and MF were effective in controlling plaque and gingivitis. WJ is significantly more effective than MF"
		BOP	S	S	S	S	S	S	
		GI	S	S	S	S	S	S	

**Group 2: waterjet to manual floss in patients with fixed orthodontics**	Sharma/<br>2008	PI<br>BOP	(2 w) S<br>S	(4 w) S<br>S	S<br>S	S<br>S	S<br>S	S<br>S	"Brushing and flossing or brushing and a WJ are effective regimens for adolescents with fixed orthodontics. The WJ was significantly better than floss for the reduction of plaque and bleeding"
	Bruce/<br>2013	PI<br>BOP	(4 w)<br>NS<br>S		NS<br>S		NS<br>NS		"The Air-Floss is as effective in reducing interproximal bleeding as flossing"
**Group 3: waterjet to manual brush alone in gingivitis or periodontist patients**	Flemmig/<br>1990	PI<br>BOP<br>GI	NA	NA	NA	NA	(3 m) NS<br>S<br>S	(6 m) NS<br>S<br>S	"WJ was effective in reducing gingivitis but not plaque score"
	Jolkovsky/<br>1990	PI<br>GI	(3 m)<br>NS<br>S		NS<br>NS		NS<br>NS		"The irrigation group improved significantly at 3 months with respect to the gingival index but not plaque index"
	Chaves/<br>1994	PI<br>BOP<br>GI	NA	NA	NA	NA	(3 m) NS<br>NS<br>NS	(6 m) NS<br>S<br>NS	"Irrigation with water shows minimal decrease in plaque, yet significantly improves clinical measures of inflammation (BOP)"
	Newman/<br>1994	PI<br>BOP<br>GI	NA		NA		(6 m) NS<br>S<br>S		"Irrigation with water in periodontal maintenance patients provided additional beneficial outcomes. These included the reduction of GI and BOP"
	Flemmig/<br>1995	PI<br>BOP<br>GI	NA		NA		(6 m) NS<br>S<br>S		"Irrigation with water in addition to regular oral hygiene can be a beneficial adjunct in patients with moderate to severe signs of periodontitis during maintenance phase"
	Cutler/<br>2000	PI<br>BOP	(2 w) S<br>S		S<br>S		S<br>S		"Oral irrigation with water had an improved therapeutic benefit for periodontitis and this improvement was accompanied by a down-

		GI	S		S		S		modulation of the pro-inflammatory cytokine profile in GCF”
	Ernst/ 2004**	PI	(4 w) S	(3 m) S	S	S		NS	“Additional use of irrigator showed no clinical benefit over routine oral hygiene in patients personally instructed and trained in oral care and who are motivated frequently”
		BOP	S	S	S	S	NA	NS	
		GI	S	S	S	S		NS	
	Akram/ 2015	PI	(3 w) S	(6 w) S	NS	NS			“Water flosser was more effective in reducing dental plaque and bleeding than brushing alone”
		BOP	S	S	NS	NS	NA	NA	
		GI	S	S	NS	S			
	Goyal/ 2018	PI	(2 w) S	(4 w) S	S	S	S	S	“Adding water flosser to manual brushing has a significant effect in reducing gingival inflammation in gingivitis patient compared to brushing alone”
		BOP	S	S	S	S	S	S	
		GI	S	S	S	S	S	S	
**Group 4: waterjet to manual brush alone in patients with fixed orthodontics**	Jackson/ 1991	PI	(4 w) NS		NS		NS		“There was no significant difference in either plaque or gingival scores for any of the methods tested”
		GI	S		S		NS		
	Burch/ 1994	PI	(4 w) S	(8 w) S	NS	S	S*	S*	“During the second month of use, the oral irrigation device, in combination with the manual toothbrush, was of significant value in reducing plaque, gingival inflammation, and bleeding”
		BOP	S	S	NS	S	S*	S*	
		GI	S	S	NS	S	S*	S*	
	Sharma/ 2008	PI	(2 w) S	(4 w) S	S	S	S	S	“Brushing and DWJ regimen was significantly better than brushing only for the reduction of plaque and bleeding”
		BOP	S	S	S	S	S	S	
	Patel/ 2015	PI	(4 w) S	(8 w) S	S	S	NS	NS	Both MB+WJ and MB alone are equally effective in plaque reduction and gingival health improvement with no significant difference between the two groups
		GI	S	S	S	S	NS	NS	

	Mazzoleni/ 2019**	PI	(4 w) NS	(6 m) NS	NS	NS	NS	NS	“WJ did not significantly improve the efficacy of routine home oral hygiene in orthodontic patients. The patient sample did not show the typical worsening of plaque and bleeding indexes, maintaining the baseline score which were originally good”
		GI	NS	NS	NS	NS	NS	NS	

BL: baseline, T1: time point 1 (duration indicated between brackets in each study), Tf: final time point in the study (duration indicated between brackets in each study), S: significant difference, NS: insignificant difference, F: facial, L: lingual, NA: p value was not mentioned in the study.

* Combined results of the 2 experimental groups (MB+WJ and PB+WJ) vs. the control group (MB alone)

** patient in all group were encouraged to use manual floss

Supplemantary Box S1: List of keywords used in the search process

**Intervention**	Waterjet, dental waterjet, WaterPik, ultra-water flosser, water flosser, water floss, water pulse, powered floss, electric floss, machined floss, oral irrigator, oral irrigation, air flosser, microdroplet device
**Control**	Dental floss, oral floss, manual floss, hand floss, waxed floss, unwaxed floss, mechanical floss, dental thread, oral thread, manual thread, hand thread, dental string, oral string, manual string, hand string, interproximal cleaning, inter-proximal cleaning, inter proximal cleaning, interproximal cleaning, interproximal cleaning, interproximal toothbrush, inter-proximal toothbrush, inter proximal toothbrush, interdental toothbrush, inter-dental toothbrush, inter dental toothbrush, interdental stimulator, inter-dental stimulator, inter dental stimulator, proxy toothbrush, proximal toothbrush, manual toothbrush, hand toothbrush, mechanical toothbrush, powered toothbrush, electric toothbrush, electronic toothbrush, sonic toothbrush

Supplemantary Box S2: Complete search strategy on MEDLINE (PubMed)

The Intervention group key words	((((((((((((((((((((((((((((((((((waterjet)) OR ((water floss))) OR ((water flosser))) OR ((water flusser))) OR ((water jet))) OR ((water irrigation))) OR ((waterpik))) OR ((water pik))) OR ((ultra water floss))) OR ((ultrawater flosser))) OR ((ultra water flosser))) OR ((water pulse))) OR ((powered dental floss))) OR ((powered oral floss))) OR ((electric dental floss))) OR ((electric oral floss))) OR ((electric floss))) OR ((powered floss))) OR ((machined floss))) OR ((machined dental floss))) OR ((machined oral floss))) OR ((machined oral floss))) OR ((microdroplet device))) OR ((oral waterjet))) OR ((dental waterjet))) OR ((dental oral irrigator))) OR ((dental oral irrigation))) OR ((oral irrigation))) OR ((dental water irrigator))) OR ((dental water irrigation))) OR ((oral water irrigator))) OR ((water irrigator))) OR ((water irrigant)))
The control group key words	(((dental floss)) OR ((oral floss))) OR ((manual floss))) OR ((hand floss))) OR ((waxed floss))) OR ((unwaxed floss))) OR ((mechanical floss))) OR ((dental thread))) OR ((oral thread))) OR ((manual thread))) OR ((interproximal cleaning))) OR ((inter proximal cleaning))) OR ((inter proximal cleaning device))) OR ((interproximal cleaning device))) OR ((inter proximal brush))) OR ((interproximal brush))) OR ((inter dental cleaning))) OR ((interdental cleaning))) OR ((inter dental cleaning device))) OR ((interdental cleaning device))) OR ((interdental brush))) OR ((inter dental brush))) OR ((inter dental toothbrush))) OR ((interdental toothbrush))) OR ((inter proximal toothbrush))) OR ((interproximal toothbrush))) OR ((toothbrush))) OR ((tooth brush))) OR ((hand toothbrush))) OR ((hand tooth brush))) OR ((manual toothbrush))) OR ((manual tooth brush))) OR ((proximal toothbrush))) OR ((proximal tooth brush))) OR ((proxy toothbrush))) OR ((proxy tooth brush))) OR ((powered toothbrush))) OR ((powered tooth brush))) OR ((electric toothbrush))) OR ((electric tooth brush))) OR ((sonic toothbrush))) OR ((sonic tooth brush)))
Combined intervention “AND” control key words	(((((((((((((((((((((((((((((((((((waterjet)) OR ((water floss))) OR ((water flosser))) OR ((water flusser))) OR ((water jet))) OR ((water irrigation))) OR ((waterpik))) OR ((water pik))) OR ((ultra water floss))) OR ((ultrawater flosser))) OR ((ultra water flosser))) OR ((water pulse))) OR ((powered dental floss))) OR ((powered oral floss))) OR ((electric dental floss))) OR ((electric oral floss))) OR ((electric floss))) OR ((powered floss))) OR ((machined floss))) OR ((machined dental floss))) OR ((machined oral floss))) OR ((machined oral floss))) OR ((microdroplet device))) OR ((oral waterjet))) OR ((dental waterjet))) OR ((dental oral irrigator))) OR ((dental oral irrigation))) OR ((oral irrigation))) OR ((dental water irrigator))) OR ((dental water irrigation))) OR ((oral water irrigator))) OR ((water irrigator))) OR ((water irrigant)))) AND ((dental floss)) OR ((oral floss))) OR ((manual floss))) OR ((hand floss))) OR ((waxed floss))) OR ((unwaxed floss))) OR ((mechanical floss))) OR ((dental thread))) OR ((oral thread))) OR ((manual thread))) OR ((interproximal cleaning))) OR ((inter

	proximal cleaning))) OR ((inter proximal cleaning device))) OR ((interproximal cleaning device))) OR ((inter proximal brush))) OR ((interproximal brush))) OR ((inter dental cleaning))) OR ((interdental cleaning))) OR ((inter dental cleaning device))) OR ((interdental cleaning device))) OR ((interdental brush))) OR ((inter dental brush))) OR ((inter dental toothbrush))) OR ((interdental toothbrush))) OR ((inter proximal toothbrush))) OR ((interproximal toothbrush))) OR ((toothbrush))) OR ((tooth brush))) OR ((hand toothbrush))) OR ((hand tooth brush))) OR ((manual toothbrush))) OR ((manual tooth brush))) OR ((proximal toothbrush))) OR ((proximal tooth brush))) OR ((proxy toothbrush))) OR ((proxy tooth brush))) OR ((powered toothbrush))) OR ((powered tooth brush))) OR ((electric toothbrush))) OR ((electric tooth brush))) OR ((sonic toothbrush))) OR ((sonic tooth brush)))))
